# Nafamostat mesylate treatment in combination with favipiravir for patients critically ill with Covid-19: a case series

**DOI:** 10.1186/s13054-020-03078-z

**Published:** 2020-07-03

**Authors:** Kent Doi, Mahoko Ikeda, Naoki Hayase, Kyoji Moriya, Naoto Morimura, the COVID-UTH Study Group, Hiromu Maehara, Shunsuke Tagami, Kazutaka Fukushima, Naho Misawa, Yutaro Inoue, Hitomi Nakamura, Daisuke Takai, Mio Kurimoto, Kurato Tokunaga, Miyuki Yamamoto, Ichiro Hirayama, Ryohei Horie, Yuri Endo, Kengo Hiwatashi, Mio Shikama, Daisuke Jubishi, Yoshiaki Kanno, Koh Okamoto, Sohei Harada, Shu Okugawa, Kohei Miyazono, Yasuyuki Seto, Jun-ichiro Inoue

**Affiliations:** 1 Department of Acute Medicine, Graduate School of Medicine, The University of Tokyo, 7-3-1 Hongo, Bunkyo-ku, Tokyo, 113-0033 Japan; 2 Department of Infection Control and Prevention, The University of Tokyo Hospital, 7-3-1 Hongo, Bunkyo-ku, Tokyo, 113-8655 Japan; 3 Department of Infectious Diseases, Graduate School of Medicine, The University of Tokyo, 7-3-1 Hongo, Bunkyo-ku, Tokyo, 113-0033 Japan; 4 Department of Molecular Pathology, Graduate School of Medicine, The University of Tokyo, Tokyo, Japan; 5 Department of Gastrointestinal Surgery, Graduate School of Medicine, The University of Tokyo, Tokyo, Japan; 6 Senior Professor Office, Graduate School of Medicine, The University of Tokyo, Tokyo, Japan

Development of specific therapy against severe acute respiratory syndrome coronavirus 2 (SARS-CoV-2) is urgently required. Several drugs such as antimalarial and anti-Ebola virus drugs are under investigation for coronavirus disease 2019 (Covid-19). Transmembrane protease serine 2 (TMPRSS2) plays a crucial role for SARS-CoV-2 entry into the cytoplasm [1]. Inhibition of TMPRSS2 protease activity is assumed to prohibit viral entry of SARS-CoV-2. Through high-throughput screening of 1017 existing drugs, a clinically available serine protease inhibitor nafamostat mesylate was identified as a potent inhibitor of Middle East respiratory syndrome coronavirus entry into human epithelial cells [2]. More recently, nafamostat mesylate was shown to inhibit the entry of SARS-CoV-2 into the human epithelial cells at EC_50_ of ~ 10 nM [3, 4]. Nafamostat mesylate has been clinically used for the treatment of acute pancreatitis and disseminated intravascular coagulation in Japan. By intravenous administration, its blood concentrations are maintained at 30–240 nM, which are sufficient to block the virus entry [3]. An anti-influenza A H1N1 virus drug favipiravir exhibits antiviral activity against other RNA viruses and therefore is expected to have antiviral action against SARS-CoV-2. This drug has been approved in Japan for novel influenza virus disease.

Eleven adults with reverse transcriptase polymerase chain reaction-confirmed SARS-CoV-2 infection were admitted to the intensive care unit (ICU) at The University of Tokyo Hospital between April 6 and April 21, 2020, and treated with nafamostat mesylate in combination with favipiravir. The demographic and clinical characteristics and the laboratory and radiologic findings at ICU admission are listed in Table 1. All the patients needed oxygen therapy. Eight patients (73%) needed invasive mechanical ventilation (MV), and 3 patients (27%) needed venovenous extracorporeal membrane oxygenation (VV-ECMO).

**Table 1:** Clinical characteristics, laboratory data, and imaging results at ICU admission

Characteristic	Measurement
Age, median (IQR), years	68 (60–69)
Male, no. (%)	10 (91%)
Body weight, median (IQR), kg	71 (69–82)
Number of patients with coexisting disorders, no. (%)	
Asthma	0 (0%)
Cancer	1 (9%)
Chronic kidney disease	0 (0%)
Chronic obstructive pulmonary disease	1 (9%)
Diabetes mellitus	3 (27%)
Hypertension	4 (36%)
Duration of symptoms before admission, median (IQR), days	8 (7–11)
Number of patients with symptoms, no. (%)	
Fever	9 (82%)
Cough	5 (45%)
Shortness of breath	8 (73%)
Laboratory data	
White blood cell count, median (IQR), per mm^3^	6900 (5800–10,850)
Lymphocyte count, median (IQR), per mm^3^	851 (759–1164)
Hemoglobin, median (IQR), g/dl	15.0 (13.5–16.3)
Platelet count, median (IQR), per mm^3^	19.6 (18.6–26.4)
Lactate dehydrogenase level, median (IQR), U/l	518 (417–752)
Aspartate aminotransferase level, median (IQR), U/l	54 (49–90)
Alanine aminotransferase level, median (IQR), U/l	47 (35–58)
Serum creatinine level, median (IQR), mg/dl	0.85 (0.70–1.03)
Creatinine kinase level, median (IQR), U/l	213 (129–579)
Prothrombin time, median (IQR), international normalized ratio	1.08 (1.03–1.13)
Activated partial thromboplastin time, median (IQR), s	28.8 (28.4–32.1)
d-dimer level, median (IQR), μg/dl	1.4 (1.1–11.8)
PaO_2_/FiO_2_ ratio, median (IQR)	131 (114–198)
SOFA score, median (IQR)	3.0 (2.5–4.5)
APACHE II score, median (IQR)	14.0 (12.0–15.5)
Computed tomography findings	
Patients with consolidation, no. (%)	6 (55%)
Patients with ground-glass opacities, no. (%)	10 (91%)
Patients with pulmonary infiltration, no. (%)	2 (18%)

*APACHE* Acute Physiology and Chronic Health Evaluation, *FiO*_*2*_ fraction of inspired oxygen, *ICU* intensive care unit, *IQR* interquartile range, *PaO*_*2*_ partial pressure of arterial oxygen, *SOFA* sequential organ failure assessment

Patients received combination treatment with nafamostat mesylate [0.2 mg per kg per hour by continuous intravenous infusion, median treatment 14 days (IQR, 10 to 14 days)] and favipiravir [3600 mg on day 1 and at 1600 mg per day on day 2 and subsequently median treatment 14 days (IQR, 12 to 14 days)]. No interruption of antiviral treatment occurred due to adverse drug reactions except for one patient who developed hyperkalemia on day 9 (by nafamostat mesylate). All 11 patients had at least 33 days of hospital follow-up. As of May 22, 1 patient, who had a do-not-resuscitate order, died on ICU day 7. Seven patients were successfully weaned from MV [median duration of MV 16 days (IQR, 10 to 19 days)] and 9 and 7 patients were discharged from the ICU and the hospital, respectively (Fig. 1).

**Fig. 1:**
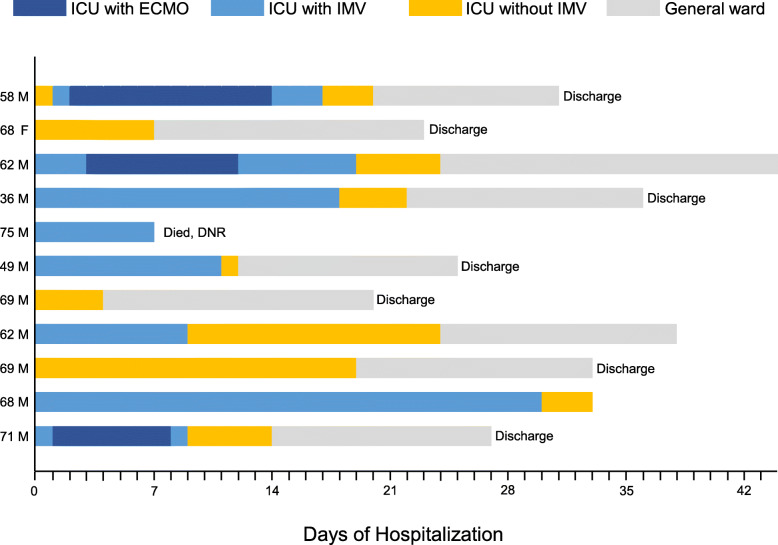
Respiratory support and outcomes of individual patients. As of May 22, 2020, 1 patient had died, while 7 patients had been extubated. All the patients receiving follow-up monitoring for at least 33 days. ICU, intensive care unit; ECMO, extracorporeal membrane oxygenation; IMV, invasive mechanical ventilation; DNR, do-not-resuscitate

This is the first report on nafamostat mesylate treatment in combination with favipiravir against Covid-19. In comparison with previous reports about critically ill patients with Covid-19, our case series also demonstrated a high number of patients (8 [73%]) who required MV requirement; however, the mortality rate was low (1 patient [9%]). Patients with severe Covid-19 often suffer from microvascular thrombosis and hemorrhage with extensive alveolar and interstitial inflammation in the lung [5]. Nafamostat mesylate might thus be effective, because it inhibits intravascular coagulopathy, in addition to directly targeting the virus entry in host epithelial cells.

In conclusion, nafamostat mesylate therapy in combination with favipiravir may allow blockade of virus entry and replication, as well as inhibition of pathogenic host response, i.e., hyper-coagulopathy. Although the number of patients in this case series was very small, this low mortality rate suggests that combination treatment of favipiravir and nafamostat mesylate may be effective for critically ill Covid-19 patients. A clinical trial for the combination treatment of nafamostat mesylate and favipiravir against Covid-19 will be initiated in Japan (jRCTs031200026).

## Data Availability

Full de-identified data of the analyses are available upon request to the corresponding author.

## Abbreviations

Covid-19: Coronavirus disease 2019
ICU: Intensive care unit
IQR: Interquartile range
MV: Mechanical ventilation
SARS-CoV-2: Severe acute respiratory syndrome coronavirus 2
TMPRSS2: Transmembrane protease serine 2
VV-ECMO: Venovenous extracorporeal membrane oxygenation
